# Can Erythropoietin Reduce Hypoxemic Neurological Damages in Neonates With Congenital Heart Defects?

**DOI:** 10.3389/fphar.2021.770590

**Published:** 2021-11-29

**Authors:** Sara Ottolenghi, Giuseppina Milano, Michele Dei Cas, Tina O. Findley, Rita Paroni, Antonio F. Corno

**Affiliations:** ^1^ Department of Health Science, University of Milan, Milan, Italy; ^2^ Department of Medicine and Surgery, University of Milano Bicocca, Milan, Italy; ^3^ Department Cœur-Vaisseaux, Cardiac Surgery Center, University Hospital of Lausanne, Lausanne, Switzerland; ^4^ Department of Pediatrics, Children’s Heart Institute, McGovern Medical School, University of Texas Health Science Center at Houston, Houston, TX, United States

**Keywords:** brain, congenital heart defects, erythropoietin, heart, hypoxemia, hypoxia inducible factors

## Abstract

Congenital heart defects (CHD), the most common cause of birth defects with increasing birth prevalence, affect nearly 1% of live births worldwide. Cyanotic CHD are characterized by hypoxemia, with subsequent reduced oxygen delivery to the brain, especially critical during brain development, beginning in the fetus and continuing through the neonatal period. Therefore, neonates with CHD carry a high risk for neurological comorbidities, even more frequently when there are associated underlying genetic disorders. We review the currently available knowledge on potential prevention strategies to reduce brain damage induced by hypoxemia during fetal development and immediately after birth, and the role of erythropoietin (EPO) as a potential adjunctive treatment. Maternal hyper-oxygenation had been studied as a potential therapeutic to improve fetal oxygenation. Despite demonstrating some effectiveness, maternal hyper-oxygenation has proven to be impractical for extensive clinical application, thus prompting the investigation of specific pathways for pharmacological intervention. Among those, the role of antioxidant pathways and Hypoxia Inducible Factors (HIF) have been studied for their involvement in the protective response to hypoxic injury. One of the proteins induced by HIF, EPO, has properties of being anti-apoptotic, antioxidant, and protective for neurons, astrocytes, and oligodendrocytes. In human trials, EPO administration in neonates with hypoxic ischemic encephalopathy (HIE) significantly reduced the neurological hypoxemic damages in several reported studies. Currently, it is unknown if the mechanisms of pathophysiology of cyanotic CHD are like HIE. Neonates with cyanotic CHD are exposed to both chronic hypoxemia and episodes of acute ischemia-reperfusion injury when undergo cardiopulmonary bypass surgery requiring aortic cross-clamp and general anesthesia. Our review supports future trials to evaluate the potential efficiency of EPO in reducing the hypoxemic neurologic damages in neonates with CHD. Furthermore, it suggests the need to identify early biomarkers of hypoxia-induced neurological damage, which must be sensitive to the neuroprotective effects of EPO.

## Introduction

Improved prenatal and neonatal detection of congenital heart defects (CHD), along with advancements in medical treatments and surgical interventions, has led to improved survival rates among children with complex CHD (Erikssen et al., 2015; Bouma and Mulder, 2017). Patients with complex CHD are at risk for neurodevelopmental impairment due to numerous biological and environmental risk factors (Marino et al., 2012). Immutable risk factors include underlying genetic conditions associated with neurodevelopmental impairment, chronic hypoxemic state, and acute reoxygenation with ischemia-reperfusion injury related to cardiopulmonary bypass. These risk factors can potentially be modified by environmental risk factors, including medical and surgical advancements to mitigate neurological insults; length and frequency of hospitalizations; education and early childhood intervention programs; and family and social support system. Still, children with complex cyanotic CHD who reach school age often require physical, occupational, and speech therapies, along with special education. Oftentimes their educational and career achievements are limited, thus impacting their quality of life (Marelli et al., 2016; Mulkey et al., 2016; Moons and Luyckx, 2019). With the increasing survival of children with CHD, research has focused on investigating the biology of the neurological insults to the brain unique to CHD, beginning in fetal development, continuing through the neonatal period and surgeries, and into childhood and later years (Miller et al., 2007; Gaynor et al., 2015). A better understanding of the biological mechanisms of injury can lead to the discovery of potential therapeutic targets aimed at improving neurological outcomes in CHD patients.

Complex cyanotic CHD are characterized by hypoxemia due to reduced oxygen delivery to the fetus during a critical period of brain development, often resulting in a smaller head circumference at birth, reflective of brain growth in infants (Williams et al., 2015; Bonthrone et al., 2021). The term hypoxemia refers to any situation in which oxygen is not available in sufficient amounts to maintain adequate cellular homeostasis. This state triggers a general organism response due to the inhibition of various oxygen-dependent enzymes. During the early 1990s, it was discovered that hypoxemia triggered the stabilization of hypoxia inducible transcription factor (HIF), allowing cells to switch from aerobic to anaerobic glycolysis (Wiesener et al., 1998). Since those early studies, the biology of the HIF pathway has been well characterized, and its role in hypoxemic injury will be outlined in further detail in this review. We will describe the known protective mechanisms in response to hypoxemic injury to the brain and preventive strategies to attenuate brain injury in the fetus and neonate. It is of growing importance the need for new pharmacological interventions, which requires, as a start, deeper knowledge of the harmful and protective biochemical pathways involved in hypoxia. One of the genes induced by *HIF2-α* (Rainville et al., 2016), *EPO* (erythropoietin)*,* is located in chromosome seven and encodes for the protein by the same name responsible for the regulation of erythropoiesis. EPO, a glycoprotein cytokine well-known for its anti-apoptotic and antioxidant role in neuronal injury (Rey et al., 2021), will be discussed in further detail as a burgeoning therapy targeting the biochemical pathways triggered by hypoxemia in the brain. In the first sections of this review, we will focus on several preclinical *in vitro* and *in vivo* studies useful in understanding the biochemical mechanisms of CHD-induced hypoxic-ischemic neurological damage. Then, we will summarize some of the main available clinical treatments and preventive strategies, together with their rationale. Finally, we will focus on the scientific evidence from *in vitro* models to clinical trials which may suggest EPO administration in CHD and non-CHD infants.

### Hypoxemia and Its Neurological Consequences

The neonatal brain requires efficient oxygen delivery as it consumes about 60% of the total body oxygen, while the adult brain only consumes about 20% (Erecinska and Silver, 1989). During embryogenesis, the heart is the first organ to form in the fetus, between the first three and 6 weeks of gestation, while the brain development continues until birth. In cardiac maldevelopment resulting in a cyanotic CHD, fetal cerebral oxygen and nutrient delivery are deficient for at least 7 months during a period critical for brain development. Clinicians and researchers are now recognizing that hypoxic tissue damage begins during fetal development, with early, postnatal/perioperative magnetic resonance imaging (MRI) studies demonstrating brain injury in the form of white matter injury or stroke in complex cyanotic CHD (Peyvandi and Donofrio, 2018). Hypoxic brain injury during fetal life correlates with impaired oxygen delivery.

Three specific congenital heart defects result in poor oxygen delivery to the brain in the fetus and neonate: 1) transposition of the great arteries, 2) “functionally” univentricular hearts, and 3) hypoplastic left heart syndrome (Sun et al., 2015; Claessens et al., 2017; Morton et al., 2017; Courtney et al., 2018; Peyvandi and Donofrio, 2018; Rychik et al., 2018; Claessens et al., 2019; Lauridsen et al., 2019). Transposition of the great arteries results in blood circulation that occurs in parallel rather than in series, necessitating deoxygenated and oxygenated blood to mix across a septal defect and patent ductus arteriosus before entering the systemic circulation. Univentricular hearts require the mixing of deoxygenated and oxygenated blood in the single ventricular chamber prior to entering the systemic circulation. In both scenarios, the brain is exposed to low oxygen delivery because of lower-than-normal oxygen saturation. In the last scenario, the left-sided heart structures are undersized including the mitral valve, left ventricle, aortic valve, and ascending aorta. The brain receives the poorest levels of oxygen delivery due to the compounded effects of low oxygen saturation and poor perfusion from limited or absent antegrade blood flow in the ascending aorta to the brain. As a result, cerebral perfusion is dependent on retrograde blood flow in the aorta of desaturated blood shunting right-to-left, from the pulmonary artery via the patent ductus arteriosus. Patients with hypoplastic left heart syndrome have the highest incidence of brain damage acquired in the fetal life when compared to the transposition of the great arteries and “functionally” univentricular hearts (Morton et al., 2015).

Following birth, neonates with cyanotic CHD often have definitive corrective surgery later in infancy for reasons such as waiting for the infant to grow and reach a size amenable to surgery or requiring surgical repair in stages. Neonates with cyanotic CHD exhibit delayed structural brain development, appearing approximately one month less mature compared to unaffected infants (Licht et al., 2009). Myelination of the cerebral cortex is a normal postnatal process beginning at 35 weeks of gestation and peaks at 1 year of age. The broad range of neurological dysfunctions documented in CHD patients is remarkably similar to the deficits observed in non-cardiac preterm survivors suffering from white matter injury, a common cause of morbidity (Wintermark et al., 2015). Preterm infants are especially highly sensitive to hyperoxia-induced oxidative stress for multiple reasons. Birth is associated with a dramatic change from an intrauterine hypoxic milieu to a relatively hyperoxic extrauterine environment, and this relative hyperoxia can be exaggerated by supplemental oxygen administration (Brill et al., 2017). Another common cause of neonatal brain injury is perinatal asphyxia-induced hypoxic ischemic encephalopathy (HIE). While HIE is the event of acute hypoxemia in a newborn who had been normally-oxygenated as a fetus, comparisons in the mechanism of brain injury have been made with cyanotic CHD. The standard of care for moderate to severe HIE involves total body cooling to 33.5°C, or therapeutic hypothermia, for 72 h in the newborn, immediately following the birth-injury (Shankaran et al., 2005; Shankaran et al., 2012). However, therapeutic hypothermia for HIE has its limitations in feasibility in resource-limited countries and has not shown to be helpful in premature infants with HIE (Bharadwaj and Bhat, 2012; Rao et al., 2017). Additionally, therapeutic hypothermia has restricted long-term benefits, as many children still suffer from long-term disability following HIE injury (Rao et al., 2017; Shankaran et al., 2017).

Erythropoietin (EPO) has long been used in preterm neonates to prevent and/or treat anemia of prematurity. While EPO treatment reduces the frequency and volume of red blood cell transfusions, its use is currently not recommended, pending the results of two ongoing clinical trials involving darbepoietin, a derivative of EPO (Ohlsson and Aher, 2017). However, it is generally well-tolerated in neonates with a reasonable safety profile. Given its neuroprotective benefits, researchers have been investigating its use in neonatal brain injury, particularly in HIE.

## The Biochemical Mechanisms of Hypoxic Damage in Heart and Brain

**FIGURE 1:**
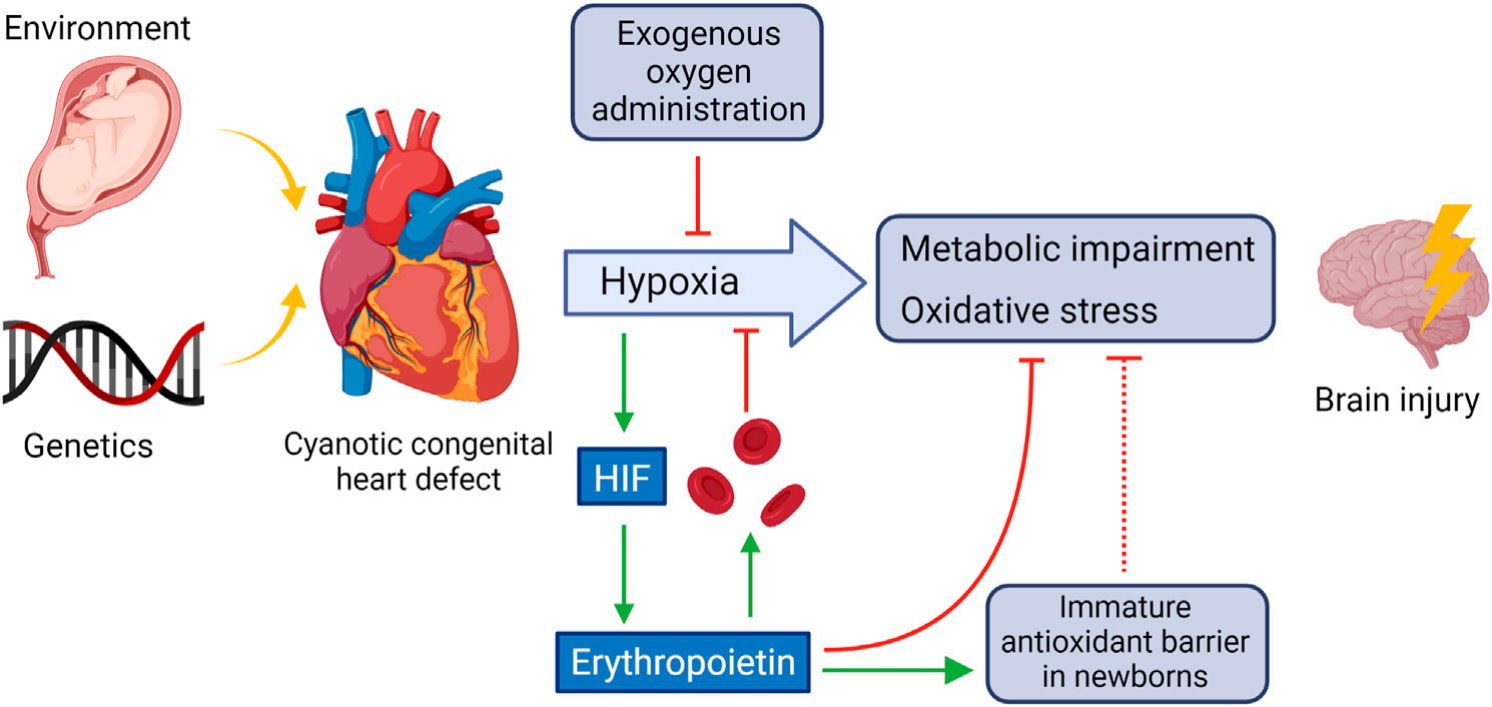
CHD-induced oxidative and hypoxic damage in the brain and potential counteractive mechanisms. HIF activated by hypoxemia triggers, among others, the synthesis of EPO which stimulates red blood cell production, in part by inhibiting apoptosis of the red blood cell precursors and helps to counteracts oxidative stress in the brain by strengthening the immature antioxidant barrier. Created using BioRender.com.

Figure 1 shows some of the main mechanisms involved in the hypoxic damage in the brain due to CHD. The main source of further damage are metabolic impairment and oxidative stress.

### HIFs

Cellular adaptive response to hypoxia is mainly orchestrated by the activation of transcription factors called hypoxia inducible factors (HIFs) (Semenza, 19852000). HIFs are heterodimeric proteins composed of oxygen-regulated HIF-1α or HIF-2α and a constitutively expressed HIF-1β subunit. Under hypoxic conditions, HIF-α proteins are stabilized for heterodimerization with HIF-β and bound to hypoxia response elements in the promoter regions of specific genes. In normoxic conditions, the fine regulation of HIF-1α is catalyzed by enzymes in the prolyl-hydroxylase domain (PHDs) that start the ubiquitination process leading to HIF-1α proteasome degradation (Semenza, 19852000). As oxygen acts as co-substrate of PHD enzymes, PHD activity decreases under hypoxic conditions, allowing HIF-α to dimerize with HIF-β and translocate to the nucleus. HIF-αβ heterodimers are then free to bind to hypoxia-response elements (HREs) within the enhancers or promoters of HIF target genes and recruit the transcriptional co-activator CBP/p300 (Semenza, 2017). This activates the transcriptional complex by promoting the regulation and expression of HIF-dependent adaptive genes that regulate diverse physiologic processes, such as angiogenesis, vascular remodeling, glucose and energy metabolism, cell proliferation and survival, and erythropoiesis and iron homeostasis (Semenza, 2017).

The understanding of these biochemical and physiological processes by HIF-1 led to the award of the 2019 Nobel Prize in Physiology or Medicine to three scientists who contributed significantly to the field, William Kaelin, Sir Peter Ratcliffe, and Gregg Semenza. Semenza’s initial work proved that HIF is a DNA-binding transcription factor for activating expression of *EPO* gene (Semenza et al., 1991). Kaelin’s group discovered that deletion of von Hippel-Lindau (VHL) results in increased expression of multiple hypoxia-induced genes, including *VEGFA* and *SLC2A1*, which encode vascular endothelial growth factor A and glucose transporter 1, respectively (Iliopoulos et al., 1996). Ratcliffe’s group confirmed the association between VHL and HIF (Maxwell et al., 1999). Their crucial discoveries and those from many other laboratories established HIF-1 as an oxygen-sensing mechanism for cells and confirmed the critical role of HIF in hypoxic cellular responses.

The three HIF isoforms (HIF-1, HIF-2, and HIF-3) have some overlapping roles but also demonstrate distinct functions in different cell types (Schödel and Ratcliffe, 2019). HIF-2α was originally named endothelial PAS domain-containing protein 1 (EPAS1), as it was found to be most strongly expressed in endothelial cells (Wiesener et al., 1998). In the kidney, HIF-2α is strongly expressed in interstitial cells, endothelial cells, and the glomeruli, but its expression is largely absent from the tubular cells, in which HIF-1α is the predominant isoform. HIF-1 and HIF-2 both transduce positive transcriptional responses to hypoxia, although their transcriptional targets, the kinetics of activation and oxygen dependence, differ. HIF-1α is induced more strongly by severe hypoxia, and its activity peaks within the first 24 h of hypoxia, whereas HIF-2α is induced by more moderate hypoxia with a prolonged activation period (Wiesener et al., 1998; Holmquist-Mengelbier et al., 2006). The HIF-3α isoform is less well-understood, in part because of its complex pattern of expression involving multiple variant transcripts derived from cell-specific patterns of alternative RNA splicing (Duan, 2016).

A growing number of pre-clinical studies in rodents suggests that the activation of HIF-1α signaling pathway prior or shortly after ischemic stroke reduces tissue damage and increases functional recovery from ischemic stroke (Ogle et al., 2012; Reischl et al., 2014; Wieronska et al., 2021). One example of an agent that stabilizes the transcriptional activator HIF-1α and activates target genes involved in compensation for ischemia are small molecule hypoxia mimics, such as vitamin E, which activates the HIF-VEGF pathway, thus increasing microvascular density, restoring local blood flow and protecting the brain from ischemic insults (Zhang et al., 2004). The stabilization of HIF-1 might be a promising therapeutic target for the treatment of neurodegenerative disorders. It has been found that iron chelators are acting against the generation of free radicals derived from iron, and also induce sufficient -but not excessive-activation of HIF-1α, so that only the hypoxia-rescue genes will be activated (Merelli et al., 2018).

The activation of HIF has been suggested to play a role in fetal development, and if timed with maternal hypoxia or placental insufficiency, may contribute to CHD morphology (Bishop and Ratcliffe, 2015; Llurba Olive et al., 2018). Additionally, HIF-1α promotes the expression of various genes to assist cells in hypoxic and ischemic conditions including vascular endothelial growth factor (*VEGF*) and *EPO*, and may directly protect neurons (Zhang et al., 2004). While HIF-1α is protective in acute hypoxia such as ischemic injury by inducing pro-survival signaling pathways, its role in chronic hypoxia is less understood (Jeewa et al., 2012; Piccoli et al., 2017). Neonates with cyanotic CHD demonstrate increased genetic expression of *HIF-1α, VEGF,* and *EPO*, in the peripheral blood, but potential effects on neuroprotection are unknown (Lemus-Varela et al., 2010).

### ROS

Under physiologic conditions, 2% of electrons involved in the mitochondrial respiratory chain react with molecular oxygen to produce reactive oxygen species (ROS). ROS generation, mainly dependent on complexes I and III, highly relies on metabolic conditions and on the intra-mitochondrial balance between oxidative and antioxidant factors (Kulkarni et al., 2007). This process, besides destroying the cell membranes, yields a large variety of toxic by-products, including β-unsaturated aldehydes (malondialdehyde, 4-hydroxy-2-nonenal) (Poli et al., 1987), that can react with carbon–carbon or carbon–oxygen double bond of proteins, DNA, RNA, glucids, altering their structure or inactivating their enzymatic function (Schaur, 2003; Forman et al., 2009).

In response to excess ROS production, antioxidant tasks are pursued by enzymes as catalases, glutathione peroxidases, thioredoxins, and peroxyredoxins. These enzymes use electron donors to tackle the further formation of ROS (Helmerhorst et al., 2015). The cell organelles peroxisomes have an important role in reducing intracellular hydrogen peroxide to avoid further formation of free radicals species. The enzyme glutathione peroxidase (GPX) on the peroxisome wall catalyses the transformation of the reduced form of the tripeptide glutathione (thiol GSH) to its oxidized form (GSSG, disulphide). Under conditions of pro- and anti-oxidant balance, up to 98% of the total glutathione pool occurs in its reduced GSH form (Owen and Butterfield, 2010). Thus, GSH/GSSG ratio is an important indicator of the cellular oxidative stress and of its compensation (Aquilano et al., 2014). EPO treatment induced time-dependent elevations of GPX in a model of neurodegenerative disease (Thompson et al., 2020).

Another important antioxidant mechanism, induced by the EPO pathway, is mediated by the enzyme superoxide dismutase (SOD), which the removal of superoxide free radicals generated from mitochondrial respiration (Muller, 2000). Overexpression of SOD1, one of the isoforms of SOD, is beneficial in case of oxidative hypoxic damage, but has no effects in the setting of neonatal hypoxia-ischemia (Sheldon et al., 2017).

Prolonged systemic hypoxia is known to elicit oxidative stress altering redox balance. Under hypoxia electron-transport rate in the mitochondria decreases, the binding of oxygen to the proteins/complexes changes, aerobic oxidative respiration is limited and partial reduction of oxygen leads to the formation of ROS. While the ischemic state induces anaerobic metabolism, a lower level of ATP production, failure of ion-exchange channels, cell swelling and impaired enzymatic activity in the cytoplasm, the reintroduction of oxygen during reperfusion exacerbates mitochondrial damage, electrolyte imbalance, and oxidative stress affecting the NADPH oxidase, nitric oxide synthase, and xanthine oxidase systems. ROS overproduction leads to cell death by different mechanisms, leading to progressive neurodegeneration (Merelli et al., 2021).

Human life *in utero* elapses in an environment that is relatively hypoxic, however, oxygen availability to the fetus is provided by adaptive mechanisms that allow oxygen delivery to tissues for physiological growth and development. The master regulator for the cell’s adaptive responses to hypoxia are HIFs, which through *VEGF* and *EPO* activation, ensure O_2_ delivery to the tissue. Vascular or metabolic alterations in the mother (preeclampsia, obesity, diabetes) causes the fetus to develop under chronic hypoxia with higher risk of developing oxidative stress that may be detrimental for its fetal and postnatal development (Torres-Cuevas et al., 2017).

Much of what is understood regarding oxygen toxicity and ROS production comes from research in preterm neonates suffering from pulmonary diseases of prematurity (e.g. bronchopulmonary dysplasia), including injury of intestinal epithelial cells (Zhao et al., 2018), and ophthalmologic and neuronal damage (Perrone et al., 2015). Hypoxia leading to asphyxia is characterized by acidosis, base deficit and lactic acidemia, and requires reoxygenation maneuvers immediately after birth for resuscitation of asphyxic neonates and preterm infants with immature lungs, surfactant production and antioxidant defense system. Attention should be paid to minimize initial damage triggered by ischemia-reoxygenation injury (Granger and Kvietys, 2015). Additionally, since the discovery of oxygen toxicity and its effects on neonatal retinal development, neonatal intensive care includes close monitoring retinal development and minimizing excessive oxygenation while providing adequate respiratory support (Tin and Gupta, 2007). Several protective strategies have been tested or hypothesized in the perinatal period, such as the administration of melatonin and therapeutic hypothermia to slow down pro-oxidant processes and oxidative stress damage (Hassell et al., 2015).

## Counteracting Hypoxemic Damage With Hyperoxia

### Maternal Hyperoxygenation

Maternal hyper-oxygenation has been studied as a potential therapeutic to improve fetal oxygenation, administered following the ultrasound diagnosis of CHD, until delivery (Co-Vu et al., 2017). The relatively small studies involved committing the mothers to several hours of oxygen administration at FiO2 of 40–60% using a face mask, until delivery. Despite evidence suggesting that the morphology improved, including pulmonary blood flow and size of left heart dimensions, the studies were highly heterogeneous in the cardiac phenotypes enrolled and protocols used, and no studies investigated the neurodevelopmental outcomes. Recently, research has gained interest in understanding the fetal cerebrovascular response to maternal hyper-oxygenation in fetuses with complex CHD (Zeng et al., 2020; Hogan et al., 2021). Still, maternal hyper-oxygenation appeared largely impractical for extensive clinical application (Rudolph, 2020), prompting the investigation of specific pathways to target pharmacological interventions.

Additionally, for the developing fetus, there are concerns regarding oxygen toxicity and radiation toxicity known to occur through a common mechanism related to increased production of ROS since the 1950s (Gerschman et al., 1954). The oxidative stress caused by ROS is a primary source of DNA damage, impairment of mitochondrial function, and organ injuries (Habre and Peták, 2014). When exposed to hyperoxia, the developing retinal endothelial cells activate a series of transcription factors including HIF-1α, insulin-like growth factor, and vascular endothelial growth factor, leading to sequelae of vessel damage and impaired vessel growth (Perrone et al., 2016). Oxidative damage may lead to the so-called “free-radical related diseases of prematurity”: bronchopulmonary dysplasia, retinopathy of prematurity, periventricularleukomalacia, intraventricular hemorrhage, oxidative hemolysis, and necrotizing enterocolitis (Perrone et al., 2015; O'Donovan and Fernandes, 2004). In the setting of maternal hyper-oxgenation supplementation, the fetus would be protected from iatrogenic diseases such a necrotizing enterocolitis (typically related to enteral feeding) or bronchopulmonary dysplasia (related to ventilator barotrauma). Whether fetuses may be at risk of injuries related to oxidative stress during maternal hyper-oxygenation warrants further investigation.

### Improving Oxygen Delivery

The formula of the oxygen delivery denotes the importance of hemoglobin concentration for optimizing oxygen delivery:

oxygen delivery=cardiac output×systemic oxygen saturation×hemoglobin

Since iron is an essential component of hemoglobin, a biological response to hypoxia is to increase the demand for iron. Guidelines for iron supplementation recommend that in iron sufficient children with hematocrits below 60%, low-dose iron supplements should be given to prevent the onset of latent iron deficiency (Kling, 2020). Iron deficiency anemia was observed as a worsening parameter in several newborns with CHD, in situations of limited resources and poor diet such as Ghana (Ossei et al., 2020).

An ineffective hematopoietic response in newborns with CHD, or ‘‘relative anemia,’’ results in polycythemia insufficient for adaptation to hypoxia. Hematocrit increases in mildly cyanotic CHD, while hemoglobin stays within the normal range. Unlike adaptations observed in animal models of chronic hypoxia, human newborns with severe CHD appear to lack a compensatory increase in hematocrit and hemoglobin, suggestive of an anemic-like state (Lim et al., 2016). On the other hand, an excessive increase in hematocrit can also impair tissue oxygen delivery due to increased blood viscosity (Linderkamp et al., 1979).

The number of transfusions received during the neonatal intensive care unit stay (especially by preterm children) also correlates with increased white matter injury observed by MRI. A reasonable “double hit” hypothesis resulting in white matter injury involves the hypoxia of low hemoglobin leading to the transfusion and the inflammation associated with transfusion (Wiesener et al., 1998).

If the formula of the oxygen delivery is considered, in the presence of neonatal hypoxemia, means to improve the oxygen delivery to the brain, and subsequently the neurological conditions include 1) increase of the cardiac output, 2) increase of the oxygen saturation. Increase in cardiac output can be achieved either with interventional catheter procedures, such as the balloon atrial septostomy (= Rashkind procedure), or with surgery, aimed at improving the intra-cardiac mixing and the effective pulmonary blood flow. Additionally, there are medications utilized to increase cardiac output and optimization of respiratory support (Tabbutt et al., 2001; Browning Carmo et al., 2007; McQuillen et al., 2007; Feltes et al., 2011). In order to increase oxygen saturation, oxygen can be administered, but excess oxygen can be a further source of oxidative stress, leading to more severe bronchopulmonary dysplasia and retinopathy of prematurity (O'Donovan and Fernandes, 2004).

## Improving Antioxidant Response Through EPO Administration

The generation of oxidative stress in various clinical situations may play an additional pivotal role in *HIF2-α* stabilization and hence EPO production. Both EPO and its receptor, primarily secreted by kidney and, in the fetal period, by the liver (Dame et al., 1998), are also detectable in the brain and upregulated during injury (Wakhloo et al., 2020). *HIF-2-α* contains an iron-responsive element in its 5’ untranslated region. Under iron-deficient conditions, when hemoglobin synthesis is decreased, *HIF2-α* translation is upregulated. These mechanisms ensure that EPO synthesis is adaptable to iron availability (Gassmann and Muckenthaler, 2015). EPO also mediates other processes such as angiogenesis, neuroprotective properties, and immune regulation.

### The EPO Related Pathway in the Developing Brain

**FIGURE 2:**
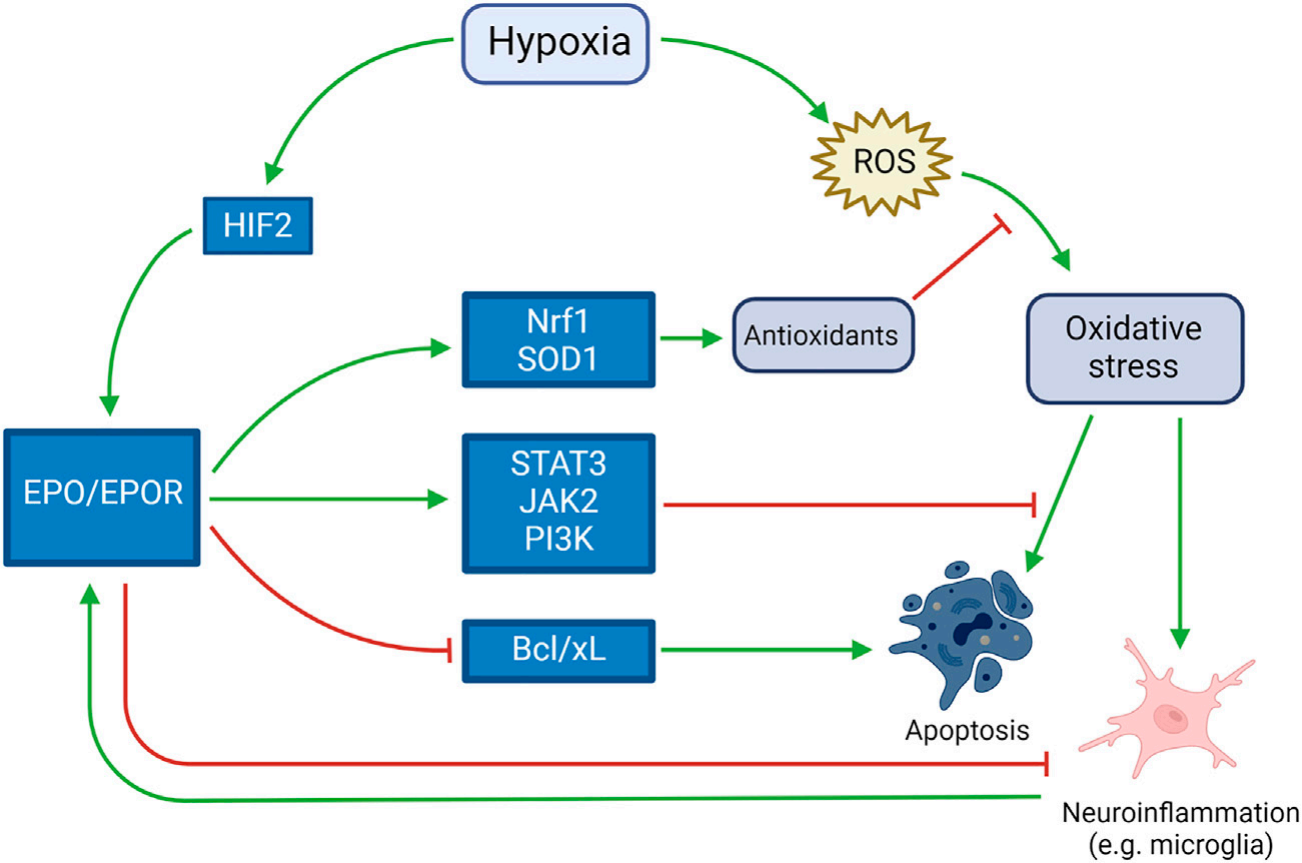
Neuroprotective role of EPO in response to hypoxia. Activated by both hypoxia itself and the harmful mechanisms induced by hypoxia (such as neuroinflammation, apoptosis and oxidative stress), EPO counteracts such mechanisms by inducing antioxidant and antiapoptotic pathways and blocking pro-apoptotic pathways.e

In the brain, investigation of the functions of EPO was initially undertaken using cultured cells before *in vivo* studies, and it is primarily produced by cultured astrocytes as a highly-specific neuronal growth factor upregulated by hypoxia (Masuda et al., 1994). Oligodendrocytes, endothelial cells, neurons, and microglia can also produce EPO when upregulated by hypoxia (van der Kooij et al., 2008). EPO and its receptor were detected in the developing brain and their persistence in the mature brain have been suggested to play a role in both neurodevelopment and homeostasis for 20 years (Juul et al., 1999). Induction of myelin genes, together with promotion of oligodendrogenesis, was theorized to play a role in EPO-induced neurological recovery in neonatal hypoxic-ischemic brain injury, where oligodendrocyte damage is an important pathogenic component (Cervellini et al., 2013). EPO receptor is present in rats’ oligodendrocytes and astrocytes in culture, and high dose EPO (1, 3, 10 U/ml) markedly enhanced the proliferation of astrocytes. These results suggested that EPO not only promotes the differentiation and/or maturation in oligodendrocytes, but also enhances the proliferation of astrocytes (Sugawa et al., 2002). The activation of EPOR downstream molecules JAK2 and PI3K, together with the regulation of the apoptotic proteins Bad and Bcl-xL, leads to a decrease in the apoptosis process (Rey et al., 2019). *In vitro*, it was observed that EPO downregulates two microRNA molecules (miR-451 and miR-855-5p) in neuroblastoma cell line. This mechanism seems to be one of the main causes for the neurotrophic, neuroprotective, antioxidant, and antiapoptotic effects (Alural et al., 2014). The antioxidant effects of EPO pathway were studied in animal models such as SOD1-overexpressing mice, that accumulate excess hydrogen peroxide, exposed to hypoxia. In situations of extreme hypoxic-ischemic damage, such study theorized that there is little chance of rescue with these neuroprotective therapies (Sheldon et al., 2017). On the other hand, when the damage is at the beginning, the damages induced by hypoxia may be counteracted by the previously described mechanisms, as shown in Figure 2.

### Neuroprotective Role of Recombinant Human EPO

A recombinant adenovirus engineered to express *HIF-1α* demonstrated that HIF-1α attenuated neuronal apoptosis partially through upregulating EPO following cerebral ischemia in a rat model (Li et al., 2020). In humans, umbilical cord blood infusion in combination with EPO administration showed therapeutic efficacy in the treatment of stroke-induced injury by promoting neurogenesis and angiogenesis through *HIF-1a* (Hwang et al., 2019).

The administration of recombinant human EPO (rhEPO) might be effective against hypoxic, ischemic, and traumatic brain injury, as well as chronic and progressive degenerative diseases (Erbayraktar et al., 2003; Brines et al., 2004; Gorio et al., 2005; Heikal et al., 2016). An increase in *BCL2* gene expression, and a decrease of ROS induced by EPO ligand antioxidant effects, activate a group of vital genes that preserve cell survival, prevent apoptotic signals, and alleviate inflammation (Castillo et al., 2018). The neuroprotective effects of EPO are related to increased resistance to oxidative stress and stabilization of the redox equilibrium, as observed in *in vitro* and *ex vivo* experiments (Castillo et al., 2018; Castillo et al., 2019; Rey et al., 2019). The effect of EPO on plasma biomarkers of brain injury in HIE patients who were randomized to receive adjunctive EPO treatment along with standard hypothermia were explored in patients enrolled in the NEATO trial (Massaro et al., 2018). While several biomarkers correlated with brain injury visualized on MRI, only Tau and brain-derived neurotrophic factor (BDNF) were associated with neurological outcomes at one year of age. In addition, EPO treatment did not appear to influence biomarkers included in the small study.

To cross the blood-brain barrier, rhEPO must be administered in high doses of 2000–5000 IU/kg of body weight (Zhang et al., 2010). The need for very high dosages to achieve neuroprotection potentially leads to elevated hematocrit levels, increased blood viscosity, and perfusion deficits in the brain. An overexpression of EPO receptor is observed after brain hypoxia, and this high expression is also needed for a successful treatment with exogenously administered high doses of rhEPO. In this regard nasal administration of lower doses of rhEPO can help to produce neuroprotection without increasing the circulating red blood cells (Merelli et al., 2011a; Merelli et al., 2015). Hypoxia may induce damage in the blood-brain barrier permitting greater penetration of EPO, and other factors, into brain tissues, where it is known to counteract oxidative damage (Castillo et al., 2018; Rey et al., 2021). One randomized clinical trial reported that low dose rhEPO intravenous treatment significantly decreased the incidence of retinopathy of prematurity, an effect of preterm hypoxia, in infant boys (Sun et al., 2020), while many other studies focused on demonstrating that such oxidative damage was not related to EPO concentrations. (Bui et al., 2021). Other clinical trials showed that EPO treatment was associated with a reduction in bronchopulmonary dysplasia, without considering the potential positive neurodevelopmental effects (Rayjada et al., 2012; Bui et al., 2019). EPO neuroprotection includes systemic effects such as enhanced erythropoiesis which increases iron utilization, thereby decreasing free iron and reducing oxidative brain injury. It has been hypothesized that systemic effects of EPO such as stabilizing oxygen availability, decreasing free iron, and reducing inflammation, complement the direct neuroprotective effects of EPO and may explain why lower dosing strategies also improve outcome (McPherson and Juul, 2010). At the same time, other forms of EPO, slightly modified to improve delivery through the blood-brain barrier, without the need of administrating high doses of EPO, are under development (Zhang et al., 2010).

## Discussion

EPO has been proposed as a potential therapeutic option in CHD in previous reviews (Stegeman et al., 2018), even if specific studies are still a limited number (Table 1).

**TABLE 1:** Studies involving EPO administration in children with CHD.

Authors	Patients (n)	Objective	EPO dosing	Outcomes
Andropoulus et al., 2013 (Andropoulos et al., 2013)	Neonates scheduled for cardiac surgery with hypothermic CPB for>60 min (n = 62)	To assess the safety and give a preliminary indication of the efficacy of EPO treatment for neuroprotection in the perioperative period for neonatal cardiac surgery	500–1,000 units/kg	No different safety profile of EPO than placebo, including major intracranial thromboses, hemorrhage, other MRI injuries, and death
Sonzogni et al., 2001 (Sonzogni et al., 2001)	Children undergoing open heart surgery (2–14 years old, n = 39)	To assess feasibility and efficacy of EPO treatment and its effect on allogenic blood transfusion in children undergoing open heart surgery	1,000 units/kg during the 3 weeks preceding operation)	EPO increases the amount of autologous blood that can be collected and minimizes allogenic blood exposure in children undergoing open heart surgery
Ootake et al., 2007 (Ootaki et al., 2007) (abstract only, no full text available)	Children undergoing cardiac surgery (cyanotic CHD, n = 10; noncyanotic CHD, n = 72)	To evaluate the efficacy of administering a single dose of rhEPO on the requirement for blood transfusion	200 units/kg plus 2 mg/kg of iron sulfate, vs 400 units/kg plus 4 mg/kg of iron sulfate, vs placebo	The administration of a single dose of rhEPO without autologous blood donations increased hematocrit levels

EPO administration to improve oxygen delivery and treat anemia in infants have been proposed by several studies, sometimes together with iron administration (Kling, 2020). A very recent meta-analysis confirmed the efficacy and safety of rhEPO in preterm infants (Qin and Qin, 2021). Some of the white matter neurological damages in such patients are quite similar to the ones observed in preterm infants (Wintermark et al., 2015), The studies investigating neurological effects of EPO in preterms might be suggestive of its potential neuroprotective effect too. Such studies are listed and commented in Table 2.

**TABLE 2:** Studies involving EPO administration in preterm infants without CHD.

Authors	Patients (n)	Objective	Role of EPO	Outcomes
Fahim et al., 2021 (Fahim et al., 2021)	Preterm infants with gestational age >30 weeks expected to survive >72 h (n = 27)	To investigate correlations between EPO concentrations and outcomes, including retinopathy of prematurity (ROP) and brain injury	Biomarker of hypoxic damage	Elevated endogenous EPO concentrations in the first 2 weeks of life are associated with lower birth weight and increased risk of adverse outcomes
			Endogenously produced	
Song et al., 2016 (Song et al., 2016)	Preterm infants (<32 weeks, n = 800)	To reduce early severe complications and improve long-term neurological outcomes in very preterm infants	Administration as drug 300-500 U/kg	EPO decreased the risk of death and moderate/severe disability at 18 months
Juul et al., 2020 (Juul et al., 2020)	Extremely preterm infants (24–27 weeks)	To prevent death and improve neurodevelopmental outcomes at 2 years of age	Administration as drug 1000 U/kg of every 48 h for a total of six doses, followed by a maintenance dose of 400 U/kg	No lower risk of death or better neurodevelopmental outcomes at 2 years of age, no detected side effects
Natalucci et al., 2016 (Natalucci et al., 2016)	Very preterm infants (26–31 weeks)	the effect of early high-dose rhEPO on the neurodevelopmental outcome at 2 years	Administration as drug 3000 IU/kg for a total of five doses	No statistically significant differences in neurodevelopmental outcomes at 2 years
Natalucci et al., 2020 (Natalucci et al., 2020)	2-year-olds who had been born very preterm (ie, <32 weeks’ gestation, n = 448)	To assess neurodevelopmental outcomes at 5 years	3000 IU/kg intravenously vs saline within 3, at 12–18, and at 36–42 postnatal hours	No lower risk of death or better neurodevelopmental outcomes at 5 years of age, no detected side effects

Recently, EPO was studied in clinical trials as a single treatment (in countries with limited access to hypothermia therapy), as adjunctive treatment to hypothermia therapy, and in preterm infants who do not qualify for hypothermia therapy (Zhu et al., 2009; Wu et al., 2016; Juul et al., 2018; Juul et al., 2020). While EPO did not reduce severe neurodevelopmental impairment or death in preterm infants with HIE, results from trials involving EPO as monotherapy and combined with therapeutic hypothermia look promising (Oorschot et al., 2020). Importantly, based on the relative successes of the clinical trials for HIE patients, the neuroprotective role of erythropoietin for children with CHD undergoing surgery justifies further investigations since studies of modifiable perioperative strategies have failed to show significant changes in the risk of poor outcomes (Marino et al., 2012). An option to increase efficacy and and avoid side effects could be intranasal administration, as suggested by *in vivo* animal studies in adult rats (Merelli et al., 2011b).

## Conclusion

Neurological damages in children with CHD are associated with elevated mortality and morbidity, seriously affecting the patients, their families, the care providers, and society at large. In this review available knowledge on potential prevention and reduction of the severity of neurological damages due to hypoxemia have been analyzed, in particular the potential role of EPO administration. Several *in vitro* and *in vivo* studies suggest the neuroprotective effects of both hypoxia-induced and administrated EPO in infants. The studies published so far suggest the need for further clinical investigations to evaluate the potential efficiency of EPO administration in reducing the hypoxemic neurologic damages in neonates with CHD.

## References

[B1] Alural B. Duran G. A. Tufekci K. U. Allmer J. Onkal Z. Tunali D. (2014). EPO Mediates Neurotrophic, Neuroprotective, Anti-oxidant, and Anti-apoptotic Effects via Downregulation of miR-451 and miR-885-5p in SH-Sy5y Neuron-like Cells. Front. Immunol. 5, 475. 10.3389/fimmu.2014.00475 25324845 PMC4179732

[B2] Andropoulos D. B. Brady K. Easley R. B. Dickerson H. A. Voigt R. G. Shekerdemian L. S. (2013). Erythropoietin Neuroprotection in Neonatal Cardiac Surgery: a Phase I/II Safety and Efficacy Trial. J. Thorac. Cardiovasc. Surg. 146 (1), 124–131. 10.1016/j.jtcvs.2012.09.046 23102686 PMC3579008

[B3] Aquilano K. Baldelli S. Ciriolo M. R. (2014). Glutathione: New Roles in Redox Signaling for an Old Antioxidant. Front. Pharmacol. 5, 196. 10.3389/fphar.2014.00196 25206336 PMC4144092

[B4] Bharadwaj S. K. Bhat B. V. (2012). Therapeutic Hypothermia Using Gel Packs for Term Neonates with Hypoxic Ischaemic Encephalopathy in Resource-Limited Settings: a Randomized Controlled Trial. J. Trop. Pediatr. 58 (5), 382–388. 10.1093/tropej/fms005 22396230

[B5] Bishop T. Ratcliffe P. J. (2015). HIF Hydroxylase Pathways in Cardiovascular Physiology and Medicine. Circ. Res. 117 (1), 65–79. 10.1161/CIRCRESAHA.117.305109 26089364 PMC4501273

[B6] Bonthrone A. F. Dimitrova R. Chew A. Kelly C. J. Cordero-Grande L. Carney O. (2021). Individualized Brain Development and Cognitive Outcome in Infants with Congenital Heart Disease. Brain Commun. 3 (2), fcab046. 10.1093/braincomms/fcab046 33860226 PMC8032964

[B7] Bouma B. J. Mulder B. J. (2017). Changing Landscape of Congenital Heart Disease. Circ. Res. 120 (6), 908–922. 10.1161/CIRCRESAHA.116.309302 28302739

[B8] Brill C. Scheuer T. Bührer C. Endesfelder S. Schmitz T. (2017). Corrigendum: Oxygen Impairs Oligodendroglial Development via Oxidative Stress and Reduced Expression of HIF-1α. Sci. Rep. 7, 46800. 10.1038/srep46800 28497799 PMC5427461

[B9] Brines M. Grasso G. Fiordaliso F. Sfacteria A. Ghezzi P. Fratelli M. (2004). Erythropoietin Mediates Tissue protection through an Erythropoietin and Common Beta-Subunit Heteroreceptor. Proc. Natl. Acad. Sci. U S A. 101 (41), 14907–14912. 10.1073/pnas.0406491101 15456912 PMC522054

[B10] Browning Carmo K. A. Barr P. West M. Hopper N. W. White J. P. Badawi N. (2007). Transporting Newborn Infants with Suspected Duct Dependent Congenital Heart Disease on Low-Dose Prostaglandin E1 without Routine Mechanical Ventilation. Arch. Dis. Child. Fetal Neonatal. Ed. 92 (2), F117–F119. 10.1136/adc.2006.096305 16905574 PMC2675450

[B11] Bui K. C. T. Ellenhorn N. Abbasi A. Villosis M. F. B. Nguyen M. Truong H. (2021). Erythropoietin Is Not a Risk Factor for Severe Retinopathy of Prematurity Among High Risk Preterm Infants. Early Hum. Dev. 161, 105440. 10.1016/j.earlhumdev.2021.105440 34407495

[B12] Bui K. C. T. Kim R. Abbasi A. Nguyen M. Villosis M. F. Chen Q. (2019). Erythropoietin Treatment Is Associated with a Reduction in Moderate to Severe Bronchopulmonary Dysplasia in Preterm Infants. A Regional Retrospective Study. Early Hum. Dev. 137, 104831. 10.1016/j.earlhumdev.2019.104831 31374455

[B13] Castillo C. Fernández-Mendívil C. Buendia I. Saavedra P. Meza C. Parra N. C. (2019). Neuroprotective Effects of EpoL against Oxidative Stress Induced by Soluble Oligomers of Aβ Peptide. Redox Biol. 24, 101187. 10.1016/j.redox.2019.101187 30965198 PMC6454060

[B14] Castillo C. Zaror S. Gonzalez M. Hidalgo A. Burgos C. F. Cabezas O. I. (2018). Neuroprotective Effect of a New Variant of Epo Nonhematopoietic against Oxidative Stress. Redox Biol. 14, 285–294. 10.1016/j.redox.2017.09.010 28987867 PMC5975214

[B15] Cervellini I. Annenkov A. Brenton T. Chernajovsky Y. Ghezzi P. Mengozzi M. (2013). Erythropoietin (EPO) Increases Myelin Gene Expression in CG4 Oligodendrocyte Cells through the Classical EPO Receptor. Mol. Med. 19, 223–229. 10.2119/molmed.2013.00013 23821361 PMC3769529

[B16] Claessens N. H. P. Chau V. de Vries L. S. Jansen N. J. G. Au-Young S. H. Stegeman R. (2019). Brain Injury in Infants with Critical Congenital Heart Disease: Insights from Two Clinical Cohorts with Different Practice Approaches. J. Pediatr. 215, 75–e2. 10.1016/j.jpeds.2019.07.017 31451185

[B17] Claessens N. H. P. Kelly C. J. Counsell S. J. Benders M. J. N. L. (2017). Neuroimaging, Cardiovascular Physiology, and Functional Outcomes in Infants with Congenital Heart Disease. Dev. Med. Child. Neurol. 59 (9), 894–902. 10.1111/dmcn.13461 28542743

[B18] Co-Vu J. Lopez-Colon D. Vyas H. V. Weiner N. DeGroff C. (2017). Maternal Hyperoxygenation: A Potential Therapy for Congenital Heart Disease in the Fetuses? A Systematic Review of the Current Literature. Echocardiography 34 (12), 1822–1833. 10.1111/echo.13722 29287137

[B19] Courtney J. A. Cnota J. F. Jones H. N. (2018). The Role of Abnormal Placentation in Congenital Heart Disease; Cause, Correlate, or Consequence? Front. Physiol. 9, 1045. 10.3389/fphys.2018.01045 30131711 PMC6091057

[B20] Dame C. Fahnenstich H. Freitag P. Hofmann D. Abdul-Nour T. Bartmann P. (1998). Erythropoietin mRNA Expression in Human Fetal and Neonatal Tissue. Blood 92 (9), 3218–3225. 10.1182/blood.v92.9.3218 9787158

[B21] Duan C. (2016). Hypoxia-inducible Factor 3 Biology: Complexities and Emerging Themes. Am. J. Physiol. Cel Physiol 310 (4), C260–C269. 10.1152/ajpcell.00315.2015 26561641

[B22] Erbayraktar S. Grasso G. Sfacteria A. Xie Q. W. Coleman T. Kreilgaard M. (2003). Asialoerythropoietin Is a Nonerythropoietic Cytokine with Broad Neuroprotective Activity *In Vivo* . Proc. Natl. Acad. Sci. U S A. 100 (11), 6741–6746. 10.1073/pnas.1031753100 12746497 PMC164517

[B23] Erecinska M. Silver I. A. (1989). ATP and Brain Function. J. Cereb. Blood Flow Metab. 9 (1), 2–19. 2642915 10.1038/jcbfm.1989.2

[B24] Erikssen G. Liestøl K. Seem E. Birkeland S. Saatvedt K. J. Hoel T. N. (2015). Achievements in Congenital Heart Defect Surgery: a Prospective, 40-year Study of 7038 Patients. Circulation 131 (4), 337–346. 10.1161/CIRCULATIONAHA.114.012033 25538230

[B25] Fahim N. M. Georgieff M. K. Zhang L. Naisbitt S. Rao R. B. Inder T. E. (2021). Endogenous Erythropoietin Concentrations and Association with Retinopathy of Prematurity and Brain Injury in Preterm Infants. PLoS One 16 (6), e0252655. 10.1371/journal.pone.0252655 34077474 PMC8171927

[B26] Feltes T. F. Bacha E. Beekman R. H. Cheatham J. P. Feinstein J. A. Gomes A. S. (2011). Indications for Cardiac Catheterization and Intervention in Pediatric Cardiac Disease: a Scientific Statement from the American Heart Association. Circulation 123 (22), 2607–2652. 10.1161/CIR.0b013e31821b1f10 21536996

[B27] Forman H. J. Zhang H. Rinna A. (2009). Glutathione: Overview of its Protective Roles, Measurement, and Biosynthesis. Mol. Aspects Med. 30 (1-2), 1–12. 10.1016/j.mam.2008.08.006 18796312 PMC2696075

[B28] Gassmann M. Muckenthaler M. U. (2015). Adaptation of Iron Requirement to Hypoxic Conditions at High Altitude. J. Appl. Physiol. (1985) 119 (12), 1432–1440. 10.1152/japplphysiol.00248.2015 26183475

[B29] Gaynor J. W. Stopp C. Wypij D. Andropoulos D. B. Atallah J. Atz A. M. (2015). Neurodevelopmental Outcomes after Cardiac Surgery in Infancy. Pediatrics 135 (5), 816–825. 10.1542/peds.2014-3825 25917996 PMC4533222

[B30] Gerschman R. Gilbert D. L. Nye S. W. Dwyer P. Fenn W. O. (1954). Oxygen Poisoning and X-Irradiation: a Mechanism in Common. Science 119 (3097), 623–626. 10.1126/science.119.3097.623 13156638

[B31] Gorio A. Madaschi L. Di Stefano B. Carelli S. Di Giulio A. M. De Biasi S. (2005). Methylprednisolone Neutralizes the Beneficial Effects of Erythropoietin in Experimental Spinal Cord Injury. Proc. Natl. Acad. Sci. U S A. 102 (45), 16379–16384. 10.1073/pnas.0508479102 16260722 PMC1283477

[B32] Granger D. N. Kvietys P. R. (2015). Reperfusion Injury and Reactive Oxygen Species: The Evolution of a Concept. Redox Biol. 6, 524–551. 10.1016/j.redox.2015.08.020 26484802 PMC4625011

[B33] Habre W. Peták F. (2014). Perioperative Use of Oxygen: Variabilities across Age. Br. J. Anaesth. 113 (2), ii26–36. 10.1093/bja/aeu380 25498579

[B34] Hassell K. J. Ezzati M. Alonso-Alconada D. Hausenloy D. J. Robertson N. J. (2015). New Horizons for Newborn Brain protection: Enhancing Endogenous Neuroprotection. Arch. Dis. Child. Fetal Neonatal. Ed. 100 (6), F541–F552. 10.1136/archdischild-2014-306284 26063194 PMC4680177

[B35] Heikal L. Ghezzi P. Mengozzi M. Stelmaszczuk B. Feelisch M. Ferns G. A. (2016). Erythropoietin and a Nonerythropoietic Peptide Analog Promote Aortic Endothelial Cell Repair under Hypoxic Conditions: Role of Nitric Oxide. Hypoxia (Auckl) 4, 121–133. 10.2147/HP.S104377 27800514 PMC5085277

[B36] Helmerhorst H. J. Schultz M. J. van der Voort P. H. de Jonge E. van Westerloo D. J. (2015). Bench-to-bedside Review: the Effects of Hyperoxia during Critical Illness. Crit. Care 19, 284. 10.1186/s13054-015-0996-4 26278383 PMC4538738

[B37] Hogan W. J. Moon-Grady A. J. Zhao Y. Cresalia N. M. Nawaytou H. Quezada E. (2021). Fetal Cerebrovascular Response to Maternal Hyperoxygenation in Congenital Heart Disease: Effect of Cardiac Physiology. Ultrasound Obstet. Gynecol. 57 (5), 769–775. 10.1002/uog.22024 32202689 PMC7673795

[B38] Holmquist-Mengelbier L. Fredlund E. Löfstedt T. Noguera R. Navarro S. Nilsson H. (2006). Recruitment of HIF-1alpha and HIF-2alpha to Common Target Genes Is Differentially Regulated in Neuroblastoma: HIF-2alpha Promotes an Aggressive Phenotype. Cancer Cell 10 (5), 413–423. 10.1016/j.ccr.2006.08.026 17097563

[B39] Hwang S. Choi J. Kim M. (2019). Combining Human Umbilical Cord Blood Cells with Erythropoietin Enhances Angiogenesis/Neurogenesis and Behavioral Recovery after Stroke. Front. Neurol. 10, 357. 10.3389/fneur.2019.00357 31024439 PMC6467968

[B40] Iliopoulos O. Levy A. P. Jiang C. Kaelin W. G. Goldberg M. A. (1996). Negative regulation of hypoxia-inducible genes by the von Hippel-Lindau protein. Proc. Natl. Acad. Sci. U S A. 93 (20), 10595–10599. 10.1073/pnas.93.20.10595 8855223 PMC38198

[B41] Jeewa A. Manickaraj A. K. Mertens L. Manlhiot C. Kinnear C. Mondal T. (2012). Genetic Determinants of Right-Ventricular Remodeling after Tetralogy of Fallot Repair. Pediatr. Res. 72 (4), 407–413. 10.1038/pr.2012.95 22797143

[B42] Juul S. E. Comstock B. A. Heagerty P. J. Mayock D. E. Goodman A. M. Hauge S. (2018). High-Dose Erythropoietin for Asphyxia and Encephalopathy (HEAL): A Randomized Controlled Trial - Background, Aims, and Study Protocol. Neonatology 113 (4), 331–338. 10.1159/000486820 29514165 PMC5980685

[B43] Juul S. E. Comstock B. A. Wadhawan R. Mayock D. E. Courtney S. E. Robinson T. (2020). A Randomized Trial of Erythropoietin for Neuroprotection in Preterm Infants. N. Engl. J. Med. 382 (3), 233–243. 10.1056/NEJMoa1907423 31940698 PMC7060076

[B44] Juul S. E. Yachnis A. T. Rojiani A. M. Christensen R. D. (1999). Immunohistochemical Localization of Erythropoietin and its Receptor in the Developing Human Brain. Pediatr. Dev. Pathol. 2 (2), 148–158. 10.1007/s100249900103 9949221

[B45] Kling P. J. (2020). Iron Nutrition, Erythrocytes, and Erythropoietin in the NICU: Erythropoietic and Neuroprotective Effects. Neoreviews 21 (2), e80–e88. 10.1542/neo.21-2-e80 32005718

[B46] Kulkarni A. C. Kuppusamy P. Parinandi N. (2007). Oxygen, the lead Actor in the Pathophysiologic Drama: Enactment of the trinity of Normoxia, Hypoxia, and Hyperoxia in Disease and Therapy. Antioxid. Redox Signal. 9 (10), 1717–1730. 10.1089/ars.2007.1724 17822371

[B47] Lauridsen M. H. Uldbjerg N. Petersen O. B. Vestergaard E. M. Matthiesen N. B. Henriksen T. B. (2019). Fetal Heart Defects and Measures of Cerebral Size. J. Pediatr. 210, 146–153. 10.1016/j.jpeds.2019.02.042 30961987

[B48] Lemus-Varela M. L. Flores-Soto M. E. Cervantes-Munguía R. Torres-Mendoza B. M. Gudiño-Cabrera G. Chaparro-Huerta V. (2010). Expression of HIF-1 Alpha, VEGF and EPO in Peripheral Blood from Patients with Two Cardiac Abnormalities Associated with Hypoxia. Clin. Biochem. 43 (3), 234–239. 10.1016/j.clinbiochem.2009.09.022 19804771

[B49] Li J. Tao T. Xu J. Liu Z. Zou Z. Jin M. (2020). HIF-1α A-ttenuates N-euronal A-poptosis by U-pregulating EPO E-xpression F-ollowing C-erebral I-schemia-reperfusion I-njury in a R-at MCAO M-odel. Int. J. Mol. Med. 45 (4), 1027–1036. 10.3892/ijmm.2020.4480 32124933 PMC7053873

[B50] Licht D. J. Shera D. M. Clancy R. R. Wernovsky G. Montenegro L. M. Nicolson S. C. (2009). Brain Maturation Is Delayed in Infants with Complex Congenital Heart Defects. J. Thorac. Cardiovasc. Surg. 137 (3), 529–537. 10.1016/j.jtcvs.2008.10.025 19258059 PMC2701902

[B51] Lim J. M. Kingdom T. Saini B. Chau V. Post M. Blaser S. (2016). Cerebral Oxygen Delivery Is Reduced in Newborns with Congenital Heart Disease. J. Thorac. Cardiovasc. Surg. 152 (4), 1095–1103. 10.1016/j.jtcvs.2016.05.027 27349283

[B52] Linderkamp O. Klose H. J. Betke K. Brodherr-Heberlein S. Bühlmeyer K. Kelson S. (1979). Increased Blood Viscosity in Patients with Cyanotic Congenital Heart Disease and Iron Deficiency. J. Pediatr. 95 (4), 567–569. 10.1016/s0022-3476(79)80770-2 480036

[B53] Llurba Olive E. Xiao E. Natale D. R. Fisher S. A. (2018). Oxygen and Lack of Oxygen in Fetal and Placental Development, Feto-Placental Coupling, and Congenital Heart Defects. Birth Defects Res. 110 (20), 1517–1530. 10.1002/bdr2.1430 30576091

[B54] Marelli A. Miller S. P. Marino B. S. Jefferson A. L. Newburger J. W. (2016). Brain in Congenital Heart Disease across the Lifespan: The Cumulative Burden of Injury. Circulation 133 (20), 1951–1962. 10.1161/CIRCULATIONAHA.115.019881 27185022 PMC5519142

[B55] Marino B. S. Lipkin P. H. Newburger J. W. Peacock G. Gerdes M. Gaynor J. W. (2012). Neurodevelopmental Outcomes in Children with Congenital Heart Disease: Evaluation and Management: a Scientific Statement from the American Heart Association. Circulation 126 (9), 1143–1172. 10.1161/CIR.0b013e318265ee8a 22851541

[B56] Massaro A. N. Wu Y. W. Bammler T. K. Comstock B. Mathur A. McKinstry R. C. (2018). Plasma Biomarkers of Brain Injury in Neonatal Hypoxic-Ischemic Encephalopathy. J. Pediatr. 194, 67–e1. 10.1016/j.jpeds.2017.10.060 29478510

[B57] Masuda S. Okano M. Yamagishi K. Nagao M. Ueda M. Sasaki R. (1994). A Novel Site of Erythropoietin Production. Oxygen-dependent Production in Cultured Rat Astrocytes. J. Biol. Chem. 269 (30), 19488–19493. 10.1016/s0021-9258(17)32195-6 8034718

[B58] Maxwell P. H. Wiesener M. S. Chang G. W. Clifford S. C. Vaux E. C. Cockman M. E. (1999). The Tumour Suppressor Protein VHL Targets Hypoxia-Inducible Factors for Oxygen-dependent Proteolysis. Nature 399 (6733), 271–275. 10.1038/20459 10353251

[B59] McPherson R. J. Juul S. E. (2010). Erythropoietin for Infants with Hypoxic-Ischemic Encephalopathy. Curr. Opin. Pediatr. 22 (2), 139–145. 10.1097/MOP.0b013e328336eb57 20090525 PMC2879270

[B60] McQuillen P. S. Nishimoto M. S. Bottrell C. L. Fineman L. D. Hamrick S. E. Glidden D. V. (2007). Regional and central Venous Oxygen Saturation Monitoring Following Pediatric Cardiac Surgery: Concordance and Association with Clinical Variables. Pediatr. Crit. Care Med. 8 (2), 154–160. 10.1097/01.PCC.0000257101.37171.BE 17273125

[B61] Merelli A. Caltana L. Girimonti P. Ramos A. J. Lazarowski A. Brusco A. (2011). Recovery of Motor Spontaneous Activity after Intranasal Delivery of Human Recombinant Erythropoietin in a Focal Brain Hypoxia Model Induced by CoCl2 in Rats. Neurotox Res. 20 (2), 182–192. 10.1007/s12640-010-9233-8 21116766

[B62] Merelli A. Caltana L. Lazarowski A. Brusco A. (2011). Experimental Evidence of the Potential Use of Erythropoietin by Intranasal Administration as a Neuroprotective Agent in Cerebral Hypoxia. Drug Metabol Drug Interact 26 (2), 65–69. 10.1515/DMDI.2011.007 21756166

[B63] Merelli A. Czornyj L. Lazarowski A. (2015). Erythropoietin as a New Therapeutic Opportunity in Brain Inflammation and Neurodegenerative Diseases. Int. J. Neurosci. 125 (11), 793–797. 10.3109/00207454.2014.989321 25405533

[B64] Merelli A. Repetto M. Lazarowski A. Auzmendi J. (2021). Hypoxia, Oxidative Stress, and Inflammation: Three Faces of Neurodegenerative Diseases. J. Alzheimers Dis. 82 (s1), S109–S126. 10.3233/JAD-201074 33325385

[B65] Merelli A. Rodríguez J. C. G. Folch J. Regueiro M. R. Camins A. Lazarowski A. (2018). Understanding the Role of Hypoxia Inducible Factor during Neurodegeneration for New Therapeutics Opportunities. Curr. Neuropharmacol 16 (10), 1484–1498. 10.2174/1570159X16666180110130253 29318974 PMC6295932

[B66] Miller S. P. McQuillen P. S. Hamrick S. Xu D. Glidden D. V. Charlton N. (2007). Abnormal Brain Development in Newborns with Congenital Heart Disease. N. Engl. J. Med. 357 (19), 1928–1938. 10.1056/NEJMoa067393 17989385

[B67] Moons P. Luyckx K. (2019). Quality-of-life Research in Adult Patients with Congenital Heart Disease: Current Status and the Way Forward. Acta Paediatr. 108 (10), 1765–1772. 10.1111/apa.14876 31136004

[B68] Morton P. D. Ishibashi N. Jonas R. A. Gallo V. (2015). Congenital Cardiac Anomalies and white Matter Injury. Trends Neurosci. 38 (6), 353–363. 10.1016/j.tins.2015.04.001 25939892 PMC4461528

[B69] Morton P. D. Ishibashi N. Jonas R. A. (2017). Neurodevelopmental Abnormalities and Congenital Heart Disease: Insights into Altered Brain Maturation. Circ. Res. 120 (6), 960–977. 10.1161/CIRCRESAHA.116.309048 28302742 PMC5409515

[B70] Mulkey S. B. Bai S. Luo C. Cleavenger J. E. Gibson N. Holland G. (2016). School-Age Test Proficiency and Special Education after Congenital Heart Disease Surgery in Infancy. J. Pediatr. 178, 47–e1. 10.1016/j.jpeds.2016.06.063 27453376 PMC5295644

[B71] Muller F. (2000). The Nature and Mechanism of Superoxide Production by the Electron Transport Chain: Its Relevance to Aging. J. Am. Aging Assoc. 23 (4), 227–253. 10.1007/s11357-000-0022-9 23604868 PMC3455268

[B72] Natalucci G. Latal B. Koller B. Rüegger C. Sick B. Held L. (2016). Effect of Early Prophylactic High-Dose Recombinant Human Erythropoietin in Very Preterm Infants on Neurodevelopmental Outcome at 2 years: A Randomized Clinical Trial. JAMA 315 (19), 2079–2085. 10.1001/jama.2016.5504 27187300

[B73] Natalucci G. Latal B. Koller B. Rüegger C. Sick B. Held L. (2020). Neurodevelopmental Outcomes at Age 5 Years after Prophylactic Early High-Dose Recombinant Human Erythropoietin for Neuroprotection in Very Preterm Infants. JAMA 324 (22), 2324–2327. 10.1001/jama.2020.19395 33289818 PMC7724553

[B74] O'Donovan D. J. Fernandes C. J. (2004). Free Radicals and Diseases in Premature Infants. Antioxid. Redox Signal. 6 (1), 169–176. 10.1089/152308604771978471 14713348

[B75] Ogle M. E. Gu X. Espinera A. R. Wei L. (2012). Inhibition of Prolyl Hydroxylases by Dimethyloxaloylglycine after Stroke Reduces Ischemic Brain Injury and Requires Hypoxia Inducible Factor-1α. Neurobiol. Dis. 45 (2), 733–742. 10.1016/j.nbd.2011.10.020 22061780 PMC3286647

[B76] Ohlsson A. Aher S. M. (2017). Early Erythropoiesis-Stimulating Agents in Preterm or Low Birth Weight Infants. Cochrane Database Syst. Rev. 11, CD004863. 10.1002/14651858.CD004863.pub5 29145693 PMC6486170

[B77] Oorschot D. E. Sizemore R. J. Amer A. R. (2020). Treatment of Neonatal Hypoxic-Ischemic Encephalopathy with Erythropoietin Alone, and Erythropoietin Combined with Hypothermia: History, Current Status, and Future Research. Int. J. Mol. Sci. 21 (4). 10.3390/ijms21041487 PMC7073127 32098276

[B78] Ootaki Y. Yamaguchi M. Yoshimura N. Oka S. Yoshida M. Hasegawa T. (2007). The Efficacy of Preoperative Administration of a Single Dose of Recombinant Human Erythropoietin in Pediatric Cardiac Surgery. Heart Surg. Forum 10 (2), E115–E119. 10.1532/HSF98.20061183 17597033

[B79] Ossei I. Buabeng K. O. Ossei P. P. S. Nguah S. B. Ayibor W. G. Anto B. P. (2020). Iron-deficiency Anaemia in Children with Congenital Heart Diseases at a Teaching Hospital in Ghana. Heliyon 6 (2), e03408. 10.1016/j.heliyon.2020.e03408 32095651 PMC7033342

[B80] Owen J. B. Butterfield D. A. (2010). Measurement of Oxidized/reduced Glutathione Ratio. Methods Mol. Biol. 648, 269–277. 10.1007/978-1-60761-756-3_18 20700719

[B81] Perrone S. Bracciali C. Di Virgilio N. Buonocore G. (2016). Oxygen Use in Neonatal Care: A Two-Edged Sword. Front. Pediatr. 4, 143. 10.3389/fped.2016.00143 28119904 PMC5220090

[B82] Perrone S. Tataranno L. M. Stazzoni G. Ramenghi L. Buonocore G. (2015). Brain Susceptibility to Oxidative Stress in the Perinatal Period. J. Matern. Fetal Neonatal. Med. 28, 2291–2295. 10.3109/14767058.2013.796170 23968388

[B83] Peyvandi S. Donofrio M. T. (2018). Circulatory Changes and Cerebral Blood Flow and Oxygenation during Transition in Newborns with Congenital Heart Disease. Semin. Pediatr. Neurol. 28, 38–47. 10.1016/j.spen.2018.05.005 30522727

[B84] Piccoli M. Conforti E. Varrica A. Ghiroldi A. Cirillo F. Resmini G. (2017). NEU3 Sialidase Role in Activating HIF-1α in Response to Chronic Hypoxia in Cyanotic Congenital Heart Patients. Int. J. Cardiol. 230, 6–13. 10.1016/j.ijcard.2016.12.123 28038803

[B85] Poli G. Albano E. Dianzani M. U. (1987). The Role of Lipid Peroxidation in Liver Damage. Chem. Phys. Lipids 45 (2-4), 117–142. 10.1016/0009-3084(87)90063-6 3319224

[B86] Qin N. Qin H. (2021). Efficacy and Safety of High and Low Dose Recombinant Human Erythropoietin on Neurodevelopment of Premature Infants: A Meta-Analysis. Medicine (Baltimore) 100 (18), e25805. 10.1097/MD.0000000000025805 33950982 PMC8104141

[B87] Rainville N. Jachimowicz E. Wojchowski D. M. (2016). Targeting EPO and EPO Receptor Pathways in Anemia and Dysregulated Erythropoiesis. Expert Opin. Ther. Targets 20 (3), 287–301. 10.1517/14728222.2016.1090975 26419263 PMC4829957

[B88] Rao R. Trivedi S. Vesoulis Z. Liao S. M. Smyser C. D. Mathur A. M. (2017). Safety and Short-Term Outcomes of Therapeutic Hypothermia in Preterm Neonates 34-35 Weeks Gestational Age with Hypoxic-Ischemic Encephalopathy. J. Pediatr. 183, 37–42. 10.1016/j.jpeds.2016.11.019 27979578 PMC5367984

[B89] Rayjada N. Barton L. Chan L. S. Plasencia S. Biniwale M. Bui K. C. (2012). Decrease in Incidence of Bronchopulmonary Dysplasia with Erythropoietin Administration in Preterm Infants: a Retrospective Study. Neonatology 102 (4), 287–292. 10.1159/000341615 22922736

[B90] Reischl S. Li L. Walkinshaw G. Flippin L. A. Marti H. H. Kunze R. (2014). Inhibition of HIF Prolyl-4-Hydroxylases by FG-4497 Reduces Brain Tissue Injury and Edema Formation during Ischemic Stroke. PLoS One 9 (1), e84767. 10.1371/journal.pone.0084767 24409307 PMC3883663

[B91] Rey F. Balsari A. Giallongo T. Ottolenghi S. Di Giulio A. M. Samaja M. (2019). Erythropoietin as a Neuroprotective Molecule: An Overview of its Therapeutic Potential in Neurodegenerative Diseases. ASN Neuro 11, 1759091419871420. 10.1177/1759091419871420 31450955 PMC6712762

[B92] Rey F. Ottolenghi S. Giallongo T. Balsari A. Martinelli C. Rey R. (2021). Mitochondrial Metabolism as Target of the Neuroprotective Role of Erythropoietin in Parkinson's Disease. Antioxidants (Basel) 10 (1). 10.3390/antiox10010121 PMC7830512 33467745

[B93] Rudolph A. M. (2020). Maternal Hyperoxygenation for the Human Fetus: Should Studies Be Curtailed? Pediatr. Res. 87 (4), 630–633. 10.1038/s41390-019-0604-4 31600768

[B94] Rychik J. Goff D. McKay E. Mott A. Tian Z. Licht D. J. (2018). Characterization of the Placenta in the Newborn with Congenital Heart Disease: Distinctions Based on Type of Cardiac Malformation. Pediatr. Cardiol. 39 (6), 1165–1171. 10.1007/s00246-018-1876-x 29728721 PMC6096845

[B95] Schaur R. J. (2003). Basic Aspects of the Biochemical Reactivity of 4-hydroxynonenal. Mol. Aspects Med. 24 (4-5), 149–159. 10.1016/s0098-2997(03)00009-8 12892992

[B96] Schödel J. Ratcliffe P. J. (2019). Mechanisms of Hypoxia Signalling: New Implications for Nephrology. Nat. Rev. Nephrol. 15 (10), 641–659. 10.1038/s41581-019-0182-z 31488900

[B97] Semenza G. L. (2017). A Compendium of Proteins that Interact with HIF-1α. Exp. Cel Res 356 (2), 128–135. 10.1016/j.yexcr.2017.03.041 PMC5541399 28336293

[B98] Semenza G. L. (19852000). HIF-1: Mediator of Physiological and Pathophysiological Responses to Hypoxia. J. Appl. Physiol. (1985) 88 (4), 1474–1480. 10.1152/jappl.2000.88.4.1474 10749844

[B99] Semenza G. L. Nejfelt M. K. Chi S. M. Antonarakis S. E. (1991). Hypoxia-inducible Nuclear Factors Bind to an Enhancer Element Located 3' to the Human Erythropoietin Gene. Proc. Natl. Acad. Sci. U S A. 88 (13), 5680–5684. 10.1073/pnas.88.13.5680 2062846 PMC51941

[B100] Shankaran S. Laptook A. R. Ehrenkranz R. A. Tyson J. E. McDonald S. A. Donovan E. F. (2005). Whole-body Hypothermia for Neonates with Hypoxic-Ischemic Encephalopathy. N. Engl. J. Med. 353 (15), 1574–1584. 10.1056/NEJMcps050929 16221780

[B101] Shankaran S. Laptook A. R. Pappas A. McDonald S. A. Das A. Tyson J. E. (2017). Effect of Depth and Duration of Cooling on Death or Disability at Age 18 Months Among Neonates with Hypoxic-Ischemic Encephalopathy: A Randomized Clinical Trial. JAMA 318 (1), 57–67. 10.1001/jama.2017.7218 28672318 PMC5793705

[B102] Shankaran S. Pappas A. McDonald S. A. Vohr B. R. Hintz S. R. Yolton K. (2012). Childhood Outcomes after Hypothermia for Neonatal Encephalopathy. N. Engl. J. Med. 366 (22), 2085–2092. 10.1056/NEJMoa1112066 22646631 PMC3459579

[B103] Sheldon R. A. Windsor C. Lee B. S. Arteaga Cabeza O. Ferriero D. M. (2017). Erythropoietin Treatment Exacerbates Moderate Injury after Hypoxia-Ischemia in Neonatal Superoxide Dismutase Transgenic Mice. Dev. Neurosci. 39 (1-4), 228–237. 10.1159/000472710 28445874 PMC5972513

[B104] Song J. Sun H. Xu F. Kang W. Gao L. Guo J. (2016). Recombinant Human Erythropoietin Improves Neurological Outcomes in Very Preterm Infants. Ann. Neurol. 80 (1), 24–34. 10.1002/ana.24677 27130143 PMC5084793

[B105] Sonzogni V. Crupi G. Poma R. Annechino F. Ferri F. Filisetti P. (2001). Erythropoietin Therapy and Preoperative Autologous Blood Donation in Children Undergoing Open Heart Surgery. Br. J. Anaesth. 87 (3), 429–434. 10.1093/bja/87.3.429 11517127

[B106] Stegeman R. Lamur K. D. van den Hoogen A. Breur J. M. P. J. Groenendaal F. Jansen N. J. G. (2018). Neuroprotective Drugs in Infants with Severe Congenital Heart Disease: A Systematic Review. Front. Neurol. 9, 521. 10.3389/fneur.2018.00521 30018590 PMC6037764

[B107] Sugawa M. Sakurai Y. Ishikawa-Ieda Y. Suzuki H. Asou H. (2002). Effects of Erythropoietin on Glial Cell Development; Oligodendrocyte Maturation and Astrocyte Proliferation. Neurosci. Res. 44 (4), 391–403. 10.1016/s0168-0102(02)00161-x 12445627

[B108] Sun H. Song J. Kang W. Wang Y. Sun X. Zhou C. (2020). Effect of Early Prophylactic Low-Dose Recombinant Human Erythropoietin on Retinopathy of Prematurity in Very Preterm Infants. J. Transl Med. 18 (1), 397. 10.1186/s12967-020-02562-y 33076939 PMC7574422

[B109] Sun L. Macgowan C. K. Sled J. G. Yoo S. J. Manlhiot C. Porayette P. (2015). Reduced Fetal Cerebral Oxygen Consumption Is Associated with Smaller Brain Size in Fetuses with Congenital Heart Disease. Circulation 131 (15), 1313–1323. 10.1161/CIRCULATIONAHA.114.013051 25762062 PMC4398654

[B110] Tabbutt S. Ramamoorthy C. Montenegro L. M. Durning S. M. Kurth C. D. Steven J. M. (2001). Impact of Inspired Gas Mixtures on Preoperative Infants with Hypoplastic Left Heart Syndrome during Controlled Ventilation. Circulation 104 (12 Suppl. 1), I159–I164. 10.1161/hc37t1.094818 11568049

[B111] Thompson A. M. Farmer K. Rowe E. M. Hayley S. (2020). Erythropoietin Modulates Striatal Antioxidant Signalling to Reduce Neurodegeneration in a Toxicant Model of Parkinson's Disease. Mol. Cel Neurosci 109, 103554. 10.1016/j.mcn.2020.103554 32971229

[B112] Tin W. Gupta S. (2007). Optimum Oxygen Therapy in Preterm Babies. Arch. Dis. Child. Fetal Neonatal. Ed. 92 (2), F143–F147. 10.1136/adc.2005.092726 17337663 PMC2675464

[B113] Torres-Cuevas I. Parra-Llorca A. Sánchez-Illana A. Nuñez-Ramiro A. Kuligowski J. Cháfer-Pericás C. (2017). Oxygen and Oxidative Stress in the Perinatal Period. Redox Biol. 12, 674–681. 10.1016/j.redox.2017.03.011 28395175 PMC5388914

[B114] van der Kooij M. A. Groenendaal F. Kavelaars A. Heijnen C. J. van Bel F. (2008). Neuroprotective Properties and Mechanisms of Erythropoietin in *In Vitro* and *In Vivo* Experimental Models for Hypoxia/ischemia. Brain Res. Rev. 59 (1), 22–33. 10.1016/j.brainresrev.2008.04.007 18514916

[B115] Wakhloo D. Scharkowski F. Curto Y. Javed Butt U. Bansal V. Steixner-Kumar A. A. (2020). Functional Hypoxia Drives Neuroplasticity and Neurogenesis via Brain Erythropoietin. Nat. Commun. 11 (1), 1313. 10.1038/s41467-020-15041-1 32152318 PMC7062779

[B116] Wieronska J. M. Cieslik P. Kalinowski L. (2021). Nitric Oxide-dependent Pathways as Critical Factors in the Consequences and Recovery after Brain Ischemic Hypoxia. Biomolecules 11 (8), 1097. 10.3390/biom11081097 34439764 PMC8392725

[B117] Wiesener M. S. Turley H. Allen W. E. Willam C. Eckardt K. U. Talks K. L. (1998). Induction of Endothelial PAS Domain Protein-1 by Hypoxia: Characterization and Comparison with Hypoxia-Inducible Factor-1alpha. Blood 92 (7), 2260–2268. 10.1182/blood.v92.7.2260.2260_2260_2268 9746763

[B118] Williams I. A. Fifer W. P. Andrews H. (2015). Fetal Growth and Neurodevelopmental Outcome in Congenital Heart Disease. Pediatr. Cardiol. 36 (6), 1135–1144. 10.1007/s00246-015-1132-6 25753684 PMC5289064

[B119] Wintermark P. Lechpammer M. Kosaras B. Jensen F. E. Warfield S. K. (2015). Brain Perfusion Is Increased at Term in the White Matter of Very Preterm Newborns and Newborns with Congenital Heart Disease: Does This Reflect Activated Angiogenesis? Neuropediatrics 46 (5), 344–351. 10.1055/s-0035-1563533 26340765

[B120] Wu Y. W. Mathur A. M. Chang T. McKinstry R. C. Mulkey S. B. Mayock D. E. (2016). High-Dose Erythropoietin and Hypothermia for Hypoxic-Ischemic Encephalopathy: A Phase II Trial. Pediatrics 137 (6). 10.1542/peds.2016-0191 27244862

[B121] Zeng S. Zhou J. Peng Q. Deng W. Zhou Q. (2020). Cerebral Hemodynamic Response to Short-Term Maternal Hyperoxygenation in Fetuses with Borderline Small Left Hearts. BMC Pregnancy Childbirth 20 (1), 411. 10.1186/s12884-020-03103-7 32680473 PMC7368676

[B122] Zhang B. Tanaka J. Yang L. Yang L. Sakanaka M. Hata R. (2004). Protective Effect of Vitamin E against Focal Brain Ischemia and Neuronal Death through Induction of Target Genes of Hypoxia-Inducible Factor-1. Neuroscience 126 (2), 433–440. 10.1016/j.neuroscience.2004.03.057 15207361

[B123] Zhang F. Xing J. Liou A. K. Wang S. Gan Y. Luo Y. (2010). Enhanced Delivery of Erythropoietin across the Blood-Brain Barrier for Neuroprotection against Ischemic Neuronal Injury. Transl Stroke Res. 1 (2), 113–121. 10.1007/s12975-010-0019-3 20577577 PMC2888513

[B124] Zhao M. Tang S. Xin J. Wei Y. Liu D. (2018). Reactive Oxygen Species Induce Injury of the Intestinal Epithelium during Hyperoxia. Int. J. Mol. Med. 41 (1), 322–330. 10.3892/ijmm.2017.3247 29138796 PMC5746288

[B125] Zhu C. Kang W. Xu F. Cheng X. Zhang Z. Jia L. (2009). Erythropoietin Improved Neurologic Outcomes in Newborns with Hypoxic-Ischemic Encephalopathy. Pediatrics 124 (2), e218–26. 10.1542/peds.2008-3553 19651565

